# Supplementary material for: The Lived Experience Of Participants in an African RandomiseD trial (LEOPARD): protocol for an in-depth qualitative study within a multisite randomised controlled trial for HIV-associated cryptococcal meningitis
Source: BMJ Open. 2021 Apr 5;11(4):e039191. doi: 10.1136/bmjopen-2020-039191 (PMC8030472; doi:10.1136/bmjopen-2020-039191)
Supplement: Supplementary data [file bmjopen-2020-039191supp001.pdf]

The Lived Experience Of Participants in an African RandomiseD controlled trial (LEOPARD)

Participant In-depth Interview Schedule

Note: This is purely a guide for a semi-structured interview and is not a rigid script. The interview should attempt to cover the key themes of enquiry outlined below but the participant should be able to steer the conversation and deviate from these themes if desired.

Introduction:

- General purpose and overview of the study
- Aims of interview
- Why the participant's cooperation is important
- Assurance of confidentiality
- What will happen with the collected information
- Any questions?
- Consent

'The aim of this exercise and series of questions is to understand a little about you and to hear your experience of the trial process from before you were recruited, the consent process, and throughout the trial itself'

Demographics and Background

- Age
- Gender
- Occupation

'At this point I would like you to consider drawing your experience in the trial as a timeline onto this piece of paper. I would like to know how you experienced each of the parts of the trial, one after the other, from just before you joined the trial until today.'

The participant can decline this approach if they wish. If they do want to draw a timeline let them direct the conversation and try to understand their recollection of events. Use prompts to ask follow-up questions as suggested below.

Before the study

- Previous experience with clinical trials, if any
- Previously held perceptions of clinical trials
- General health
- Circumstances in which participant became unwell
- How dis/orientated they were, their recollection of events
- The admission to the hospital, including experience of diagnostic lumbar puncture
- Diagnosis of cryptococcal meningitis and any other illness

Recruitment

- Experience of being approached by the team

LEOPARD Participant Interview Schedule: Version 1.0 (28th June 2018)

- First impressions of the clinical trial
- Thoughts on the participant information sheet

Consent

- How did they decide
- What was their motivation and what were their main concerns
- How long did it take to decide
- Did they feel under pressure to consent and if so, by whom
- With whom did they decide
- Did someone decide on their behalf and if so, what is their recollection of that and how did they feel both around that time and now
- When completing the form did they feel that they knew what they were signing up for
- Is there any way this process could have been improved

Within the trial (inpatient)

- Was anything different after they entered the trial compared to before
- What did they think about the nature, number and frequency of the procedures they had e.g. blood tests and lumbar punctures
- What did they think about the drugs they were receiving particularly the night time doses
- Are there any specific experiences whilst in hospital they would like to discuss
- Were they confused by what was going on at any point
- How was the communication and care from the trial team

Within the trial (outpatient)

- How was the outpatient clinic and did you have any concerns (such as confidentiality, security, cleanliness)
- How was your experience of those outpatient visits
- Did they miss any appointments during the trial and if so, why
- At any time did they consider leaving the trial and if so, why
- What did they think about the transport reimbursement, was it enough, did it play a role in encouraging them to attend outpatient visits
- How did they feel being asked the health economics questions
- Can you summarise the AMBITION trial

For participants that were confused, ask these questions at appropriate moments

- Did they understand what was happening
- If not, when did they begin to understand what was happening
- Did their confusion resolve all at once or did it come and go
- When they were informed they were in a clinical trial, what were their thoughts
- Who provided consent for them when they were confused, have they discussed this with the person/people and how do they feel about this now

For participants that have completed the study, ask these questions at appropriate moments

- How do they feel now they have left the trial
- Have they been back to their usual care provider and if so, how was that experience

LEOPARD Participant Interview Schedule: Version 1.0 (28th June 2019)

- What would they like to have seen done differently within the course of the trial
- If they were approached to take part in a clinical trial in the future what would they do and why

Closing:

Is there anything else you think is important that we have not talked about?

- Summarise
- Thank participant
- Provide contacts to participant

Second interview

A second interview will take place after the participant has exited the AMBITION study. During the second interview, spend time reviewing the information that was captured in the first and asking the participant if they have changed how they feel since exiting the study.

Any aspects of this interview schedule that were not captured in the first interview can be addressed in the second.

Lawrence DS, *et al. BMJ Open* 2021; 11:e039191. doi: 10.1136/bmjopen-2020-039191
