# Supplementary material for: The Lived Experience Of Participants in an African RandomiseD trial (LEOPARD): protocol for an in-depth qualitative study within a multisite randomised controlled trial for HIV-associated cryptococcal meningitis
Source: BMJ Open. 2021 Apr 5;11(4):e039191. doi: 10.1136/bmjopen-2020-039191 (PMC8030472; doi:10.1136/bmjopen-2020-039191)
Supplement: Supplementary data [file bmjopen-2020-039191supp002.pdf]

The Lived Experience Of Participants in an African RandomiseD controlled trial (LEOPARD)

Next-of-kin In-depth Interview Schedule

Note: This is purely a guide for a semi-structured interview and is not a rigid script. The interview should attempt to cover the key themes of enquiry outlined below but the participant should be able to steer the conversation and deviate from these themes if desired.

Introduction:

- General purpose and overview of the study
- Aims of interview
- Why the participant's cooperation is important
- Assurance of confidentiality
- What will happen with the collected information
- Any questions?
- Consent

'The aim of this exercise and series of questions is to understand a little about you and to hear your experience as the next-of-kin of someone who was recruited into the AMBITION trial. We are interested to hear your experience of the trial process for your loved one from before they were recruited, the consent process, and throughout the trial itself'

Demographics and Background

- Age
- Gender
- Occupation

'At this point I would like you to consider drawing your experience with the trial as a timeline onto this piece of paper. I would like to know how you experienced each of the parts of the trial, one after the other, from just before you were aware of the trial until today.'

The next-of-kin participant can decline this approach if they wish. If they do want to draw a timeline let them direct the conversation and try to understand their recollection of events. Use prompts to ask follow-up questions as suggested below.

Before the study

- Previous experience with clinical trials, if any
- Previously held perceptions of clinical trials
- Circumstances in which the trial participant became unwell
- How dis/orientated they felt to be at the time
- The admission to the hospital, including experience of diagnostic lumbar puncture
- How and if they were informed of the diagnosis of cryptococcal meningitis and any other illness

Recruitment

LEOPARD Next-of-kin Interview Schedule: Version 1.0 (28th June 2019)

- Experience of being approached by the team
- First impressions of the clinical trial
- Thoughts on the participant information sheet

Consent (Next-of-kin perspective)

- How did they decide
- What was their motivation and what were their main concerns
- How long did it take to decide
- Did they feel under pressure to consent and if so, by whom
- With whom did they decide
- When completing the form did they feel that they knew what they were signing their loved one up for
- Is there any way this process could have been improved

Consent (Participant perspective)

- Did they think their loved one understood what was happening
- If not, when did they begin to understand what was happening
- Did their confusion resolve all at once or did it come and go
- When they were informed they were in a clinical trial, were they part of the re-consent process
- Have they ever discussed this issue of consent with their loved one and if so would they be willing to share this discussion with the researcher

Within the trial (inpatient)

- Was anything different after the participant entered the trial compared to before
- What did they think about the nature, number and frequency of the procedures their loved one had e.g. blood tests and lumbar punctures
- What did they think about the drugs they were receiving particularly the night time doses
- Are there any specific experiences whilst in hospital they would like to discuss
- Were they confused by what was going on at any point
- How was the communication and care from the trial team
- Did they feel that they were involved in the trial process

For the next-of-kin that accompanied the participant to outpatient appointments, ask these questions at appropriate moments

- How was the outpatient clinic and did you have any concerns (such as confidentiality, security, cleanliness)
- How was your experience of those outpatient visits
- Did your loved one miss any appointments during the trial and if so, why
- At any time did they consider removing their loved one from the trial and if so, why
- At any time did their loved one consider removing themselves from the trial and if so, why
- What did they think about the transport reimbursement, was it enough, did it play a role in encouraging their loved one to attend outpatient visits and for them to accompany them
- Can they summarise the AMBITION trial

LEOPARD Next-of-kin Interview Schedule: Version 1.0 (28th June 2019)

Reflections on the trial

- How do they feel about the trial in general
- What would they like to have seen done differently within the course of the trial
- If they were approached to take part in a clinical trial in the future what would they do and why

Closing:

Is there anything else you think is important that we have not talked about?

- Summarise
- Thank participant
- Provide contacts to participant

LEOPARD Next-of-kin Interview Schedule: Version 1.0 (28th June 2019)
