# Supplementary material for: The Lived Experience Of Participants in an African RandomiseD trial (LEOPARD): protocol for an in-depth qualitative study within a multisite randomised controlled trial for HIV-associated cryptococcal meningitis
Source: BMJ Open. 2021 Apr 5;11(4):e039191. doi: 10.1136/bmjopen-2020-039191 (PMC8030472; doi:10.1136/bmjopen-2020-039191)
Supplement: Supplementary data [file bmjopen-2020-039191supp003.pdf]

AMBITION-*cm*
AMBIsome Therapy Induction OptimizatioN

LEOPARD

The Lived Experience Of Participants in an African RandomiseD controlled trial (LEOPARD)

Researcher In-depth Interview Schedule

Note: This is purely a guide for a semi-structured interview and is not a rigid script. The interview should attempt to cover the key themes of enquiry outlined below but the participant should be able to steer the conversation and deviate from these themes if desired.

Introduction:

- General purpose and overview of the study
- Aims of interview
- Why the participant's cooperation is important
- Assurance of confidentiality
- What will happen with the collected information
- Any questions?
- Consent

'The aim of the first series of questions is to contextualise you within the Ambition study and the clinical research community'

Demographics and Background:

- Job title and role
- Institution and number of years there
- Training / qualifications and their locations
- Research posts previously held and their locations

The Ambition study:

- How became involved
- Current role and responsibilities
- Level of engagement with trial participants

Previous research experience:

- Background of working with participants of other trials
- With individuals within Ambition and/or not affiliated
- In other institutions
- In transnational research partnerships

'Drawing predominantly on your current experience within the Ambition study but also from your previous work (if any) please can you share your thoughts on the following:'

Trial participant experience:

- General impressions of how participants experience a trial
- What you and fellow researchers are good at
- What you are not so good at
- Experience of evaluating trial participant experience

LEOPARD Researcher Interview Schedule: Version 1.0 (28th June 2019)

- Suggestions for improvements

Specifically aim to focus on: the consent process, recruiting participants with impaired consciousness and the death of participants, prompting if required. Probing to draw on specific examples to elicit narratives.
'Do you think that the issues you have brought up are specific to your hospital / institution / city / country. Where else can you see they do / may occur?'

Transnational research partnerships:

- Understanding of the EDCTP and how it works
- Perceived benefits of such an approach
- Any shortcomings
- Capacity building
- Ownership
- Impact on the global research agenda
- Any suggestions for improvement

'Do you think that the issues you have brought up are specific to your hospital / institution / city / country. Where else can you see they do / may occur?'

Closing:

Is there anything else you think is important that we have not talked about?

- Summarise
- Thank participant
- Provide contacts to participant
