# Supplementary material for: The Lived Experience Of Participants in an African RandomiseD trial (LEOPARD): protocol for an in-depth qualitative study within a multisite randomised controlled trial for HIV-associated cryptococcal meningitis
Source: BMJ Open. 2021 Apr 5;11(4):e039191. doi: 10.1136/bmjopen-2020-039191 (PMC8030472; doi:10.1136/bmjopen-2020-039191)
Supplement: Author's manuscript [file bmjopen-2020-039191.draft_revisions.pdf]

BMJ Open is committed to open peer review. As part of this commitment we make the peer review history of every article we publish publicly available.

When an article is published we post the peer reviewers' comments and the authors' responses online. We also post the versions of the paper that were used during peer review. These are the versions that the peer review comments apply to.

The versions of the paper that follow are the versions that were submitted during the peer review process. They are not the versions of record or the final published versions. They should not be cited or distributed as the published version of this manuscript.

BMJ Open is an open access journal and the full, final, typeset and author-corrected version of record of the manuscript is available on our site with no access controls, subscription charges or pay-per-view fees (http://bmjopen.bmj.com).

If you have any questions on BMJ Open's open peer review process please email info.bmjopen@bmj.com

BMJ Open

The Lived Experience Of Participants in an African RandomiseD trial (LEOPARD): Protocol for an in-depth qualitative study within a multi-site randomised controlled trial for HIV-associated cryptococcal meningitis.

Journal:	*BMJ Open*
Manuscript ID	bmjopen-2020-039191
Article Type:	Protocol
Date Submitted by the Author:	07-Apr-2020

Complete List of Authors:	Lawrence, David; Botswana-Harvard AIDS Institute Partnership; London School of Hygiene and Tropical Medicine Department of Clinical Research,<br>Tsholo, Katlego; Botswana-Harvard AIDS Institute Partnership<br>Ssali, Agnes; MRC/UVRI & LSHTM Uganda Research Unit<br>Mupambireyi, Zivai; Centre for Sexual Health and HIV/AIDS Research<br>Hoddinott, Graeme; Stellenbosch University Faculty of Medicine and Health Sciences, Desmond Tutu TB Centre<br>Nyirenda, Deborah; Malawi Liverpool Wellcome Trust Clinical Research Programme<br>Meya, David; Makerere University, Infectious Diseases Institute<br>Ndholvu, Chiratidzo; University of Zimbabwe College of Health Sciences, Department of Medicine<br>Harrison, Thomas; St George's University of London, Institute for Infection and Immunity<br>Jarvis, Joseph; Botswana-Harvard AIDS Institute Partnership; London School of Hygiene and Tropical Medicine, Department of Clinical Research<br>Seeley, Janet; MRC/UVRI & LSHTM Uganda Research Unit; London School of Hygiene and Tropical Medicine,

Keywords:	HIV & AIDS < INFECTIOUS DISEASES, Clinical trials < THERAPEUTICS, QUALITATIVE RESEARCH

SCHOLARONE™
Manuscripts

I, the Submitting Author has the right to grant and does grant on behalf of all authors of the Work (as defined in the below author licence), an exclusive licence and/or a non-exclusive licence for contributions from authors who are: i) UK Crown employees; ii) where BMJ has agreed a CC-BY licence shall apply, and/or iii) in accordance with the terms applicable for US Federal Government officers or employees acting as part of their official duties; on a worldwide, perpetual, irrevocable, royalty-free basis to BMJ Publishing Group Ltd ("BMJ") its licensees and where the relevant Journal is co-owned by BMJ to the co-owners of the Journal, to publish the Work in this journal and any other BMJ products and to exploit all rights, as set out in our licence.

The Submitting Author accepts and understands that any supply made under these terms is made by BMJ to the Submitting Author unless you are acting as an employee on behalf of your employer or a postgraduate student of an affiliated institution which is paying any applicable article publishing charge ("APC") for Open Access articles. Where the Submitting Author wishes to make the Work available on an Open Access basis (and intends to pay the relevant APC), the terms of reuse of such Open Access shall be governed by a Creative Commons licence – details of these licences and which Creative Commons licence will apply to this Work are set out in our licence referred to above.

Other than as permitted in any relevant BMJ Author's Self Archiving Policies, I confirm this Work has not been accepted for publication elsewhere, is not being considered for publication elsewhere and does not duplicate material already published. I confirm all authors consent to publication of this Work and authorise the granting of this licence.

For peer review only

The Lived Experience Of Participants in an African RandomiseD trial (LEOPARD): Protocol for an in-depth qualitative study within a multi-site randomised controlled trial for HIV-associated cryptococcal meningitis.

David S. Lawrence (david.s.lawrence@lshtm.ac.uk)[1,2†],
Katlego Tsholo (katlegotsholo@yahoo.ie)[2],
Agnes Ssali (agnes.ssali@mrcuganda.org)[3],
Zivai Mupambireyi (zietawana@yahoo.co.uk)[4],
Graeme Hoddinott (graemeh@sun.ac.za)[5],
Deborah Nyirenda (deborahchirwa@gmail.com)[6],
David Meya (david.meya@gmail.com)[7],
Chiratidzo Ndhlovu (mascen@mweb.co.zw)[8],
Thomas S. Harrison (tharriso@sgul.ac.uk)[9],
Joseph N. Jarvis (joseph.jarvis@lshtm.ac.uk)[1,2*] and
Janet Seeley (janet.seeley@lshtm.ac.uk)[1,3*]

[1] Department of Clinical Research, Faculty of Infectious and Tropical Diseases, London School of Hygiene and Tropical Medicine, London, UK
[2] Botswana-Harvard AIDS Institute Partnership, Gaborone, Botswana
[3] MRC/UVRI & LSHTM Uganda Research Unit, Entebbe, Uganda
[4] Centre for Sexual Health and HIV/AIDS Research, Harare, Zimbabwe
[5] Desmond Tutu TB Centre, Department of Paediatrics and Child Health, Faculty of Medicine and Health Sciences, Stellenbosch University, Cape Town, South Africa
[6] Malawi Liverpool Wellcome Trust Clinical Research Programme, Queen Elizabeth Central Hospital, Blantyre, Malawi
[7] Infectious Diseases Institute, Makerere University, Kampala, Uganda
[8] Department of Medicine, University of Zimbabwe College of Health Sciences, Parirenyatwa Hospital, Harare, Zimbabwe
[9] Institute for Infection and Immunity, St George's University of London, and St George's University Hospitals NHS Foundation Trust, London, UK

[†]Corresponding author: david.s.lawrence@lshtm.ac.uk, +267 72464834
[*]Equal contribution

Word count: 3486.

For peer review only

ABSTRACT

Introduction: Individuals recruited into clinical trials for life-threatening illnesses are particularly vulnerable. This is especially true in low-income settings. The decision to enrol may be influenced by existing inequalities, poor healthcare infrastructure and fear of death. Where patients are confused or unconscious the responsibility for this decision falls to relatives. This qualitative study is nested in the ongoing AMBIsome Therapy Induction OptimisatioN(AMBITION) trial. AMBITION is recruiting participants from five countries in sub-Saharan Africa and is trialling a novel treatment approach for HIV-associated cryptococcal meningitis, an infection known to affect brain function. We aim to learn from the experiences of participants, relatives and researchers involved in AMBITION.

Methods and Analysis: We will collect data through in-depth interviews with trial participants and the next-of-kin of participants who were confused at enrolment and therefore provided surrogate consent. Data will be collected in Gaborone, Botswana; Kampala, Uganda and Harare, Zimbabwe. Interviews will follow a narrative approach including participatory drawing of participation timelines. This will be supplemented by direct observation of the research process at each of the three recruiting hospitals. Interviews will also take place with researchers from the African and European institutions that form the partnership through which the trial is administered. Interviews will be transcribed verbatim, translated(if necessary), and organised thematically for narrative analysis.

Ethics and Dissemination: This study has been approved by the Human Resource Development Council, Gaborone(HPDME:13/18/1); Makerere School of Health Sciences IRB, Kampala(REF: 2019-061); University of Zimbabwe Joint Research Ethics Committee, Harare(REF: 219/19), and the London School of Hygiene and Tropical Medicine(REF: 17957). Study findings will be shared with research participants from the sites, key stakeholders at each research institution and Ministries of Health to help inform the development and implementation of future trials. The findings of this study will be published in journals and presented at academic meetings.

Trial Registration: Registered at www.clinicaltrials.gov:NCT04296292.

Keywords: HIV; cryptococcal meningitis; clinical trials; informed consent; qualitative research

For peer review only

STRENGTHS AND LIMITATIONS OF THIS STUDY

- There has been no previous qualitative study conducted in a low-income setting which has aimed to explore the experience of individuals who enrol into a clinical trial for the management of a life-threatening illness.
- We plan to collect data from trial participants, their next-of-kin and researchers working on a multi-site clinical trial and by doing this we can elicit a broad range of perspectives and experiences that can inform the improvement of this and similar trials in the future.
- This study can prospectively provide feedback to improve an ongoing clinical trial.
- By adopting a multi-site approach, we can compare and contrast experiences across different settings to understand which are shared and which are unique to a particular context.
- The study team are from multiple social and behavioural science disciplines meaning that interpretation of the data will be informed by a range of social theoretical perspectives.
- This study is taking place in a single clinical trial and will collect data from individuals in Botswana, Uganda and Zimbabwe only which means that the results may not be broadly generalisable.

For peer review only

INTRODUCTION

Since the start of the HIV epidemic our knowledge and understanding of the epidemiology and management of HIV and its numerous complications has exponentially increased. This knowledge has been produced through the conduct of clinical research which would not be possible without the willing consent of participants[1, 2]. Although ART programmes have expanded dramatically and AIDS related deaths have reduced, there were still an estimated 940,000 people who died from AIDS in 2017[3]. In individuals with advanced HIV disease the search for superior treatment options for fatal opportunistic infections continues.

The AMBITION Trial

The AMBIsome Therapy Induction OptimisatioN (AMBITION) trial is a phase-III multi-centred randomised controlled trial recruiting patients with HIV-associated cryptococcal meningitis (CM)[4] (Figure 1). CM is a fungal infection of the brain that occurs most frequently in severely immunocompromised individuals with a CD4 count of less than 100 cells/uL[5]. There are approximately 223,000 incident cases of CM globally, with 73% of these occurring in sub-Saharan Africa. Annual global deaths are estimated at 180,000 and CM is responsible for roughly 15% of all AIDS-related deaths[6]. The nature of the infection means that roughly 40% of patients present with confusion[7] and some with a significantly reduced level of consciousness.

AMBITION is testing a new treatment for CM, a single, high-dose of a less toxic, liposomal form of amphotericin, and is recruiting 850 participants from eight hospitals across five African countries: Botswana, Malawi, South Africa, Uganda and Zimbabwe. Patients consent for themselves if able and if confused or comatose then a surrogate will do so on their behalf. Participants are followed up daily during their initial inpatient admission (roughly two-weeks) and then fortnightly as an outpatient until they complete the study at ten-weeks. Participants have their medical expenses paid for and receive transport reimbursements to attend outpatient appointments. AMBITION is funded by the European and Developing Countries Clinical Trials Partnership (EDCTP) which brings together researchers from institutions in low-to-middle income countries (LMICs) and Europe.

The AMBITION trial creates a rich environment for an in-depth qualitative study for a number of reasons.

Why participants are motivated to participate in trials: In routine care, mortality with the best standard of care treatment for CM is roughly 50% at 10 weeks[8]. In recent CM trials using the same

For peer review only

regimen mortality is roughly 40%[7, 9]. It has been observed that even when using the same drugs as in routine care, trial participants often do better[8]. The reasons for this include having a dedicated research team with more time for patients, better management of drug-induced toxicities and aggressive management of raised intracranial pressure, a common and potentially fatal complication of CM, and, inevitably, some selection of trial participants. Further widening outcomes between routine and trial settings in CM, is the fact that the most effective drugs may be unavailable, or only sporadically available, in routine care. Clinical trials are however designed to answer a research question, the findings of which it is hoped will later be of benefit to a larger population. Some individuals may benefit by participating but it is not designed so that everyone will[10]. Despite this it is not uncommon for research participants to expect a personal therapeutic benefit from the treatment they receive, including in placebo-controlled trials [11, 12]. Other commonly identified motivators are material benefits including free healthcare and transport reimbursements[13-15] but altruism is also a factor[16]. In AMBITION it is fair to expect that all participants will benefit, compared to routine care. What is not understood is how this impacts both patients and researchers when it comes to motivating to enrol in the trial. Their motivation may be rooted in the economic inequality that exists between patient and research institution and which permeate the concept of voluntary participation. Voluntariness is understood as an autonomous choice without material entanglements and the principle of autonomy is often held above others when it comes to consenting for a clinical trial[17]. Research participants who lack agency are therefore subject to 'structural coercion' whereby their social and economic situation drives them into research participation as a means of navigating their illness and because they may not have any other options to get the care they need or desire[18]. This is polarised when the chance of death is high, such as in CM.

Whom to consent when the patient cannot: In the context of life-threatening illness there are questions about when to obtain consent and who to obtain it from. One option is to commence trial procedures and defer consent until the patient is stable, which was acceptable to 70% of parents in a UK-based emergency paediatric study who felt the process was too much to handle in a stressful situation[19]. These findings are consistent with other studies from the UK [20-22]. The Declaration of Helsinki states that it is acceptable to recruit someone without capacity in best interests[23] and it has been argued that by delaying treatment whilst waiting for consent both risks losing out on the potential health benefit of that specific emergency treatment but also underappreciating the impact of emergency treatment due to systematically delayed initiation[24]. An alternative is therefore to waiver informed consent completely, as was the approach in a post-partum haemorrhage trial in the

For peer review only

UK which found that the perceptions of those who gave consent, had a surrogate, or waived consent were not dissimilar [12].

Regarding who provides the consent, it is typical for surrogates to consent on behalf of an unwell patient who is confused or comatose. Within CM studies, roughly 40% of participants are confused and if they regain capacity they re-consent for themselves. Research in HICs has identified that there is generally good concordance between surrogates and patients when it comes to agreeing to consent to both real-life and hypothetical trials but that this is reduced in high-risk trials[25, 26]. In LMICs multiple actors are often involved in the consent process with partners, parents, older family members and community leaders being consulted [11, 27], particularly in the case of severe illness[14]. This extends the process of gaining consent and can delay recruitment and treatment. According to a systematic review of 21 studies in Africa, only 47% of participants undergoing informed consent understood trial procedures such as randomisation and placebo and only 30% were aware they may not experience a therapeutic benefit of participation[28]. Another review found that understanding is significantly diminished amongst those who are critically ill[29]. To date there have been no in-depth qualitative studies in LMICs exploring the process of consent from the perspective of an acutely unwell adult or their consenting next-of-kin.

Participant and Next-of-Kin Experience: We use the broad term of participant experience to encompass the way that an individual navigates through the scheduled events of a clinical trial as detailed in the protocol. Time is a prominent factor throughout this process. An illness occurs at a specific time in someone's life and the entire trial experience is time-bound and shaped by the protocolised schedule of events. A large portion of the ethnographic work exploring participant experience of research in LMICs has elicited data concerning rumours, most commonly blood stealing, which are often dismissed by researchers as expressions of ignorance but are interpreted by social scientists as forms of popular resistance[30-32]. Most ethnographic exploration of rumours has been situated in trials of healthy individuals in trials and less commonly in acute, life-threatening illness. Lumbar puncture, the procedure used to diagnose and treat CM is known to be associated with rumours of causing death[33]. This has not been extensively studied using ethnographic methods but lumbar puncture refusal is common and can be fatal.

In the United States there has been an increasing call to assess clinical trial participant 'patient satisfaction' through the use of surveys or interviews which aim to hear the participant's voice and respond by making local improvement to the trial[34]. In LMICs this approach is less common but the

For peer review only

concept of 'good participatory practice' has been developed by the WHO over the years [35] and this involves elements related to the participant experience[36]. No ethnographic work has explored these in the context of acute illness research in sub-Saharan Africa. Research within healthy volunteer studies has found that where poor outcomes such as severe disability or death occur, this has led to the apportioning of blame or the generation of rumours about research studies and institutions[30, 37]. An exploration within AMBITION, where poor outcomes are not uncommon, could provide an opportunity to inform and potentially improve the conduct of this trial and others in the future.

Researcher Experience: Paul Farmer (2002) wrote that 'researcher and subject are living in different worlds'[38] and it is commonly perceived that there is a mismatch between researcher and participant understanding of the research process[30]. Large, randomised controlled trials like AMBITION employ a large number of individuals from different countries[39]. Clinical researchers who interact with individuals and their next-of-kin throughout the trial timeline [15, 27, 40, 41] and a whole range of professionals who are well placed to comment on the research process, regulatory approvals and implementation of a trial and can provide a practical insight and suggestions for improvement [42]. As partners in the research process they can reflect on how clinical trials are conceptualised and designed in addition to the benefits and shortcomings of trans-national partnerships and how we can optimise these relationships for the benefit of participants[43]. International researchers often have a broad range of experience working in clinical trials and can reflect on the evolution of clinical trials over time. As representatives of institutions which are partners (and often the lead) on grant applications, they often help to steer the clinical trial agenda in the region and are well placed to comment on how trials can be improved.

Aim and Objectives

The aim of this study is to explore the experience of participants, their next-of-kin and researchers within the AMBITION trial. By doing this we hope to learn how we can improve the trial experience within AMBITION and future trials for life-threatening illnesses.

Our specific objectives are:

From the perspectives of the participant, next-of-kin and researcher:

1. To build an understanding of the factors that enhance or diminish a clinical trial experience.

From the perspective of the researcher:

2. To compare the individual researcher's experience of the conceptualisation, development, initiation and implementation of a multi-centred clinical trial in Africa.

For peer review only

METHODS AND ANALYSIS

Study design

An in-depth qualitative study entitled the Lived Experience of Participants in an African RandomiseD trial (LEOPARD). We will adopt a combination of in-depth interviews and direct observations to explore the experience of participants, their next-of-kin and researchers within the AMBITION trial (Figure 2).

Developing of the methodology

The LEOPARD study was conceived by DSL but the methodology was refined with the valuable input of social scientists from each of the six AMBITION trial sites. Each social scientist has a particular interest in clinical trials. Over a series of one-on-one discussions and group calls the LEOPARD study evolved. Having developed a consensus on a methodology it was necessary to determine the feasibility of collecting data from six sites. Recruitment into the LEOPARD study will take place in Gaborone, Harare and Kampala. The reason for limiting data collection to three sites is to enable in-depth data collection and to avoid simply skimming the surface by diluting down data collection across multiple sites. These three sites represent diverse HIV epidemics, healthcare systems and political contexts which can be explored during data analysis.

Conceptual framework

We will use narrative analysis to explore how the concept of time shapes the experience of a life-threatening illness and a clinical trial. Drawing on Nancy Munn's theory of temporalization we will look at how time is experienced by different individuals and how the pressure of a life-threatening illness impacts the perception of time as well as the complex decision to enrol (or not) in a clinical trial. By understanding how time and pressure impact the meaning and understanding of events at a time of crisis, we hope to learn how clinical trials can be better tailored to the needs of individuals with life-threatening illnesses. Narrative analysis is more commonly adopted by studies exploring chronic health conditions but the exploration of time is well suited to narrative analysis and a clinical trial, which has a clearly defined temporal structure, provides a rich setting for storytelling.

In-depth interviews with AMBITION trial participants

The purpose of the IDIs with AMBITION trial participants is to collect personal accounts of their experience within the trial. Individuals who were both orientated and confused at baseline will be approached but all participants will need to have decision making capacity to contribute to the IDI, meaning that those who were confused will now no longer be. We will aim to recruit a maximum of

For peer review only

20 participants from each of the three sites, 60 in total, with a proposed gender balance of 50-60% male and 40-50% female which is in line with the epidemiology of cryptococcal meningitis at the sites. Consecutively eligible individuals will be approached to participate in two IDIs. One will take place at least six weeks into the ten week AMBITION trial and the other will take place at least four weeks after the trial. The reason for this is to allow reflection on the trial when one is both within and outside of it. Interviews will follow a broad interview schedule and the participant will be invited to draw a timeline of the events before, during and after the trial.

In-depth interviews with the next-of-kin of AMBITION trial participants

The purpose of the IDIs with the next-of-kin of AMBITION trial participants is to collect personal accounts from individuals who have cared for and made important decisions about someone with a life-threatening illness. We use the term next-of-kin as a broad umbrella term to include any individual who may be the legal representative, a caregiver, or a surrogate of the participant. This individual will have provided consent for the participant to enrol into the AMBITION trial even if they may not have been the legally defined next-of-kin. We will aim to recruit a maximum of 15 individuals from each site, 45 in total, with no specification for gender. Consecutively eligible individuals will be approached to participate in a single IDI which will take place at least six weeks into the AMBITION trial. At the time of the IDI it will not be necessary for the trial participant to have regained decision-making capacity and these IDIs do not need to be linked to those with participants, although it is anticipated that some will be. Interviews will again follow a broad interview schedule and the participant will be invited to draw a timeline of the events.

In-depth interviews with AMBITION researchers

The purpose of the IDIs with AMBITION researchers is to understand their perspectives on how research is designed and implemented in Africa. Interviews will take place with researchers from each of the research institutions which form the AMBITION consortium. At African sites where trial participants are being recruited we will approach a range of individuals with different roles including senior and junior researchers, research doctors and nurses, laboratory scientists, pharmacists and study coordinators. In addition, individuals who are based at European institutions will be approached. We will aim for a maximum of 12 individuals from each of the three participating African sites and four from each of the five European sites. The maximum number of researcher interviews will be 56. Individuals will be conveniently sampled and interviewed on a single occasion, following a broad interview schedule.

For peer review only

Direct observations of AMBITION researchers

A period of 12 months will be spend conducting ethnographic fieldwork at the African sites. The objective of this work is to contextualise the data from IDIs within the broader research environment. As the primary focus is on improving the trial for participants, observations will be largely based in the clinical environment, with emphasis placed on observing clinical staff. A total of four researchers from each of the three African sites will be invited to participate in direct observations. It will be made clear that this is not a method designed to appraise an individual, but an opportunity to spend a defined period of time observing events that take place within the research process. Observations will be coupled with brief questions to those in close proximity to the activity under observation.

Principles of recruitment

Eligible individuals will be approached to enrol in the study by a social scientist. In the case of AMBITION trial participants and their next-of-kin, this will be done in the local language by an experienced social scientist at that site. In the case of AMBITION researchers this will be DSL who is part of the AMBITION Trial Management Group in his role as Lead Clinician for the trial. Eligible individuals will be provided with a Participant Information Sheet and given the opportunity to ask questions. If they agree to participate, they will sign an Informed Consent Form and given the opportunity to withdraw their consent at any time, without giving a reason. Interviews will take place in a mutually acceptable location, be recorded with a digital voice recorder and notes will be taken during the interview. Observations will not be recorded and field notes will be made after the period of observation has finished.

Confidentiality

All study documents will be kept on the person of the researcher or in a secure, locked location at all times. All digital documents will be on a password protected, encrypted computer, backed up regularly and only shared with the study team. Names of interviewees will not be used at any stage of the data collection process. Pre-determined identification numbers will be used on data collection forms. Audio recordings will not start until the interviewee has given consent and will not record their name. Pseudonyms will be used throughout. The location of researcher participants will be anonymised because the small number of eligible participants means that stating their location could make it possible to identify them.

For peer review only

Data analysis

Audio recordings will be transcribed verbatim into MS Word, translated into English in a separate second step if necessary, then exported to NVIVO-11 for coding and analysis. The first two IDIs from each group of participants will be analysed and discussed to enable iterative refinement of the data collection approach. Similarly, regular meetings will be used to review data, refine data collection tools and assess for data saturation. We will organise the data thematically and analyse it using narrative analysis at the country level by the social science team at each site. All data from AMBITION researchers will be analysed together using thematic analysis which will be performed in six phases: familiarisation with data, initial code generation, searching for themes, reviewing themes, defining and naming themes and presenting final conclusions. These analyses will then be combined in a meta-synthesis of all data, irrespective of location or informant, to identify any areas of disconnect and, by comparing with country specific analyses, to assess generalisability of findings.

Patient and Public Involvement

This protocol has been reviewed by Community Advisory Board members, expert patients and HIV activists from across the African sites. These individuals and groups will continue to be consulted throughout the data collection process and during the dissemination of research findings.

Ethics and Dissemination

This study has been approved by the Human Resource Development Council, Gaborone (HPDME:13/18/1); Makerere School of Health Sciences IRB, Kampala (REF: 2019-061); University of Zimbabwe Joint Research Ethics Committee, Harare (REF: 219/19), and the London School of Hygiene and Tropical Medicine (REF: 17957). Study findings will be shared with research participants from the African and European sites, key stakeholders at each research institution and Ministries of Health to help inform the development and implementation of future trials. The findings of this study will be published in journals and presented at academic meetings.

Authors Contributions: DSL wrote the initial manuscript and trial protocol and is the Chief Investigator for the study. KT, AS and ZM are social scientists based at each of the three African sites. GH is a social scientist in South Africa and contributed to the study design. DN is a social scientist in Malawi and contributed to the study design. DM and CN are the AMBITION and LEOPARD principal investigators at the Kampala and Harare sites respectively. TH and JNJ are co-Chief Investigators of the AMBITION study. JS and JNJ jointly supervise DSL. All authors have been actively involved in the development of

For peer review only

this study, the data collection tools and the analysis plan. All authors have reviewed and approved the final manuscript.

Funding statement: This work is funded by the UK National Health Service (NHS) National Institute for Health Research (NIHR), using Official Development Assistant (ODA) funding through a Global Health Professorship to JNJ (Grant RP-2017-08-ST2-012). The views expressed are those of the authors and not necessarily those of the NHS, NIHR, the Department of Health and Social Care, or other funding entities.

Competing Interests: None declared.

Figure 1: AMBITION Study Schema

Figure 2: LEOPARD Study Schema

For peer review only

REFERENCES

1. Broder S. The development of antiretroviral therapy and its impact on the HIV-1/AIDS pandemic. Antiviral research. 2010;85(1):1-18.

2. Epstein S. Democracy, expertise and AIDS treatment activism. In: Kleinman DL, editor. Science, Technology and Democracy. New York, USA: SUNY Press; 2000.

3. UNAIDS. Miles to go - closing gaps, breaking barriers, righting injustices. Global AIDS Update 2018. Geneva: UNAIDS; 2018 13th August 2018.

4. Lawrence DS, Youssouf N, Molloy SF, Alanio A, Alufandika M, Boulware DR, et al. AMBIsome Therapy Induction OptimisatioN (AMBITION): High Dose AmBisome for Cryptococcal Meningitis Induction Therapy in sub-Saharan Africa: Study Protocol for a Phase 3 Randomised Controlled Non-Inferiority Trial. Trials. 2018;19(1):649.

5. Lawrence DS, Youssouf N, Molloy S, Alanio A, Alufandika M, Boulware DR, et al. AMBIsome Therapy Induction OptimisatioN (AMBITION): High Dose Ambisome for Cryptococcal Meningitis Induction Therapy in sub-Saharan Africa: Study Protocol for a Phase 3 Randomised Controlled Non-Inferiority Trial. Trials. 2018;In Press.

6. Rajasingham R, Smith RM, Park BJ, Jarvis JN, Govender NP, Chiller TM, et al. Global burden of disease of HIV-associated cryptococcal meningitis: an updated analysis. The Lancet Infectious diseases. 2017;17(8):873-81.

7. Molloy SF, Kanyama C, Heyderman RS, Loyse A, Kouanfack C, Chanda D, et al. Antifungal Combinations for Treatment of Cryptococcal Meningitis in Africa. The New England journal of medicine. 2018;378(11):1004-17.

8. Tenforde MW, Gertz AM, Lawrence DS, Wills NK, Guthrie BL, Farquhar C, et al. Mortality from HIV-associated meningitis in sub-Saharan Africa: a systematic review and meta-analysis. Journal of the International AIDS Society. 2020;23(1):e25416.

9. Beardsley J, Wolbers M, Kibengo FM, Ggayi AB, Kamali A, Cuc NT, et al. Adjunctive Dexamethasone in HIV-Associated Cryptococcal Meningitis. The New England journal of medicine. 2016;374(6):542-54.

10. Molyneux CS, Peshu N, Marsh K. Understanding of informed consent in a low-income setting: three case studies from the Kenyan Coast. Soc Sci Med. 2004;59(12):2547-59.

11. Leach A, Hilton S, Greenwood BM, Manneh E, Dibba B, Wilkins A, et al. An evaluation of the informed consent procedure used during a trial of a Haemophilus influenzae type B conjugate vaccine undertaken in The Gambia, West Africa. Soc Sci Med. 1999;48(2):139-48.

12. Houghton G, Kingdon C, Dower M, Shakur-Still H, Alfirevic Z. What women think about consent to research at the time of an obstetric emergency: a qualitative study of the views of a cohort of World Maternal Antifibrinolytic Trial participants. BJOG : an international journal of obstetrics and gynaecology. 2018;125(13):1744-53.

13. Corneli AL, McKenna K, Perry B, Ahmed K, Agot K, Malamatsho F, et al. The science of being a study participant: FEM-PrEP participants' explanations for overreporting adherence to the study pills and for the whereabouts of unused pills. Journal of acquired immune deficiency syndromes (1999). 2015;68(5):578-84.

14. Gikonyo C, Bejon P, Marsh V, Molyneux S. Taking social relationships seriously: lessons learned from the informed consent practices of a vaccine trial on the Kenyan Coast. Soc Sci Med. 2008;67(5):708-20.

15. Ssali A, Poland F, Seeley J. Volunteer experiences and perceptions of the informed consent process: Lessons from two HIV clinical trials in Uganda. BMC Med Ethics. 2015;16(1):86.

16. Katz AWK, Mensch BS, Woeber K, Musara P, Etima J, van der Straten A. Understanding women's motivations to participate in MTN-003/VOICE, a phase 2b HIV prevention trial with low adherence. BMC women's health. 2019;19(1):18-.

17. Geissler PW, Kelly A, Imoukhuede B, Pool R. 'He is now like a brother, I can even give him some blood'--relational ethics and material exchanges in a malaria vaccine 'trial community' in The Gambia. Soc Sci Med. 2008;67(5):696-707.

For peer review only

18. Fisher JA. Expanding the frame of "voluntariness" in informed consent: structural coercion and the power of social and economic context. Kennedy Institute of Ethics journal. 2013;23(4):355-79.
19. Gamble C, Nadel S, Snape D, McKay A, Hickey H, Williamson P, et al. What Parents of Children Who Have Received Emergency Care Think about Deferring Consent in Randomised Trials of Emergency Treatments: Postal Survey. PloS one. 2012;7(5):e35982.
20. Kenyon S, Dixon-Woods M, Jackson CJ, Windridge K, Pitchforth E. Participating in a trial in a critical situation: a qualitative study in pregnancy. Quality & safety in health care. 2006;15(2):98-101.
21. Corrigan O. Empty ethics: the problem with informed consent. Sociology of health & illness. 2003;25(7):768-92.
22. Behrendt C, Golz T, Roesler C, Bertz H, Wunsch A. What do our patients understand about their trial participation? Assessing patients' understanding of their informed consent consultation about randomised clinical trials. J Med Ethics. 2011;37(2):74-80.
23. World Medical Association Declaration of Helsinki: ethical principles for medical research involving human subjects. Jama. 2013;310(20):2191-4.
24. Roberts I, Prieto-Merino D, Shakur H, Chalmers I, Nicholl J. Effect of consent rituals on mortality in emergency care research. Lancet (London, England). 2011;377(9771):1071-2.
25. Newman JT, Smart A, Reese TR, Williams A, Moss M. Surrogate and patient discrepancy regarding consent for critical care research. Critical care medicine. 2012;40(9):2590-4.
26. Coppolino M, Ackerson L. Do surrogate decision makers provide accurate consent for intensive care research? Chest. 2001;119(2):603-12.
27. Kingori P. Experiencing everyday ethics in context: frontline data collectors perspectives and practices of bioethics. Soc Sci Med. 2013;98:361-70.
28. Afolabi MO, Okebe JU, McGrath N, Larson HJ, Bojang K, Chandramohan D. Informed consent comprehension in African research settings. Tropical Medicine & International Health. 2014;19(6):625-42.
29. Tam NT, Huy NT, Thoa le TB, Long NP, Trang NT, Hirayama K, et al. Participants' understanding of informed consent in clinical trials over three decades: systematic review and meta-analysis. Bulletin of the World Health Organization. 2015;93(3):186-98h.
30. Fairhead J, Leach M, Small M. Where techno-science meets poverty: medical research and the economy of blood in The Gambia, West Africa. Soc Sci Med. 2006;63(4):1109-20.
31. Geissler PW. 'Kachinja Are Coming!': Encounters around Medical Research Work in a Kenyan Village. Africa: Journal of the International African Institute. 2005;75(2):173-202.
32. Geissler PW, Pool R. Editorial: Popular concerns about medical research projects in sub-Saharan Africa--a critical voice in debates about medical research ethics. Tropical medicine & international health : TM & IH. 2006;11(7):975-82.
33. Thakur KT, Mateyo K, Hachaambwa L, Kayamba V, Mallewa M, Mallewa J, et al. Lumbar puncture refusal in sub-Saharan Africa: A call for further understanding and intervention. Neurology. 2015;84(19):1988-90.
34. Pflugeisen BM, Rebar S, Reedy A, Pierce R, Amoroso PJ. Assessment of clinical trial participant patient satisfaction: a call to action. Trials. 2016;17(1):483.
35. McGrory E, Irvin A, Heise L. Research Rashomon: Lessons from the Cameroon Pre-exposure Prophylaxis Trial Site. Washington DC: Global Campaign for Microbicides at PATH; 2009.
36. Mack N, Kirkendale S, Omullo P, Odhiambo J, Ratlhagana M, Masaki M, et al. Implementing good participatory practice guidelines in the FEM-PrEP Preexposure Prophylaxis Trial for HIV Prevention among African Women: a focus on local stakeholder involvement. Open Access Journal of Clinical Trials. 2013;5:127-35.
37. Geissler PW. 'Transport to Where?': Reflections on the problem of value and time a propos an awkward practice in medical research. J Cult Econ. 2011;4(1):45-64.
38. Farmer P. Can transnational research be ethical in the developing world? Lancet (London, England). 2002;360(9342):1266.

For peer review only

39. Molyneux S, Geissler PW. Ethics and the ethnography of medical research in Africa. Soc Sci Med. 2008;67(5):685-95.
40. Kingori P, Gerrets R. Morals, morale and motivations in data fabrication: Medical research fieldworkers views and practices in two Sub-Saharan African contexts. Soc Sci Med. 2016;166:150-9.
41. Monroe A, Nakigozi G, Ddaaki W, Bazaale JM, Gray RH, Wawer MJ, et al. Qualitative insights into implementation, processes, and outcomes of a randomized trial on peer support and HIV care engagement in Rakai, Uganda. BMC infectious diseases. 2017;17(1):54.
42. Vischer N, Pfeiffer C, Limacher M, Burri C. "You can save time if..."-A qualitative study on internal factors slowing down clinical trials in Sub-Saharan Africa. PloS one. 2017;12(3):e0173796.
43. Franzen SRP, Chandler C, Enquselassie F, Siribaddana S, Atashili J, Angus B, et al. Understanding the investigators: a qualitative study investigating the barriers and enablers to the implementation of local investigator-initiated clinical trials in Ethiopia. BMJ open. 2013;3(11):e003616.

For peer review only

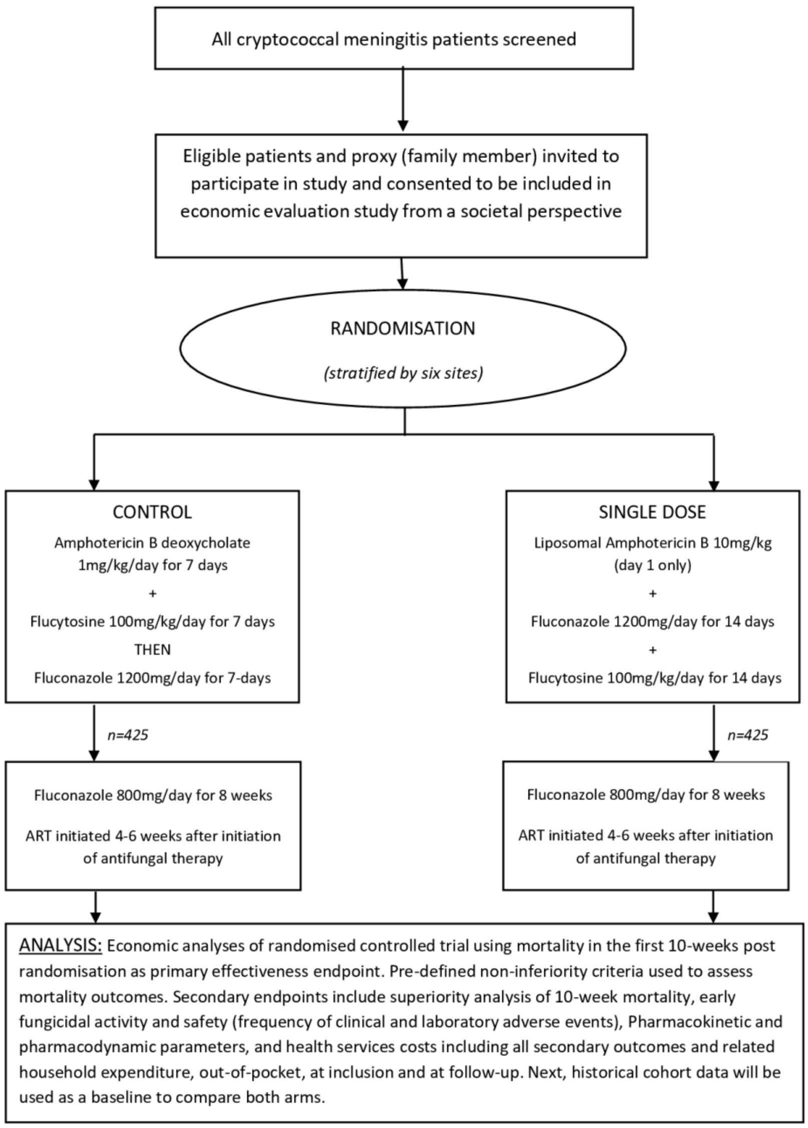

AMBITION Study Schema

117x155mm (300 x 300 DPI)

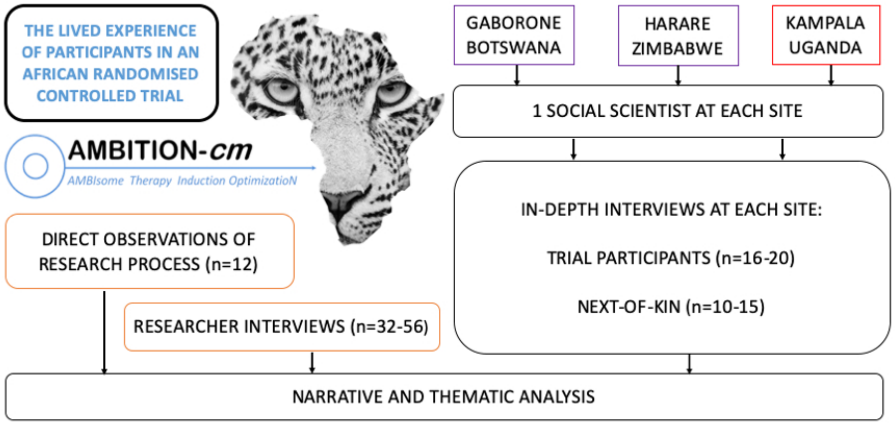

LEOPARD Study Schema

348x179mm (70 x 70 DPI)

BMJ Open

The Lived Experience Of Participants in an African RandomiseD trial (LEOPARD): Protocol for an in-depth qualitative study within a multi-site randomised controlled trial for HIV-associated cryptococcal meningitis.

Journal:	*BMJ Open*
Manuscript ID	bmjopen-2020-039191.R1
Article Type:	Protocol
Date Submitted by the Author:	12-Jan-2021
Complete List of Authors:	Lawrence, David; Botswana-Harvard AIDS Institute Partnership; London School of Hygiene and Tropical Medicine Department of Clinical Research,<br>Tsholo, Katlego; Botswana-Harvard AIDS Institute Partnership<br>Ssali, Agnes; MRC/UVRI & LSHTM Uganda Research Unit<br>Mupambireyi, Zivai; Centre for Sexual Health and HIV/AIDS Research<br>Hoddinott, Graeme; Stellenbosch University Faculty of Medicine and Health Sciences, Desmond Tutu TB Centre<br>Nyirenda, Deborah; Malawi Liverpool Wellcome Trust Clinical Research Programme<br>Meya, David; Makerere University, Infectious Diseases Institute<br>Ndhlovu, Chiratidzo; University of Zimbabwe College of Health Sciences, Department of Medicine<br>Harrison, Thomas; St George's University of London, Institute for Infection and Immunity<br>Jarvis, Joseph; Botswana-Harvard AIDS Institute Partnership; London School of Hygiene and Tropical Medicine, Department of Clinical Research<br>Seeley, Janet; MRC/UVRI & LSHTM Uganda Research Unit; London School of Hygiene and Tropical Medicine,
**Primary Subject Heading**:	Ethics
Secondary Subject Heading:	Global health, HIV/AIDS, Qualitative research, Sociology
Keywords:	HIV & AIDS < INFECTIOUS DISEASES, Clinical trials < THERAPEUTICS, QUALITATIVE RESEARCH

SCHOLARONE™
Manuscripts

I, the Submitting Author has the right to grant and does grant on behalf of all authors of the Work (as defined in the below author licence), an exclusive licence and/or a non-exclusive licence for contributions from authors who are: i) UK Crown employees; ii) where BMJ has agreed a CC-BY licence shall apply, and/or iii) in accordance with the terms applicable for US Federal Government officers or employees acting as part of their official duties; on a worldwide, perpetual, irrevocable, royalty-free basis to BMJ Publishing Group Ltd ("BMJ") its licensees and where the relevant Journal is co-owned by BMJ to the co-owners of the Journal, to publish the Work in this journal and any other BMJ products and to exploit all rights, as set out in our licence.

The Submitting Author accepts and understands that any supply made under these terms is made by BMJ to the Submitting Author unless you are acting as an employee on behalf of your employer or a postgraduate student of an affiliated institution which is paying any applicable article publishing charge ("APC") for Open Access articles. Where the Submitting Author wishes to make the Work available on an Open Access basis (and intends to pay the relevant APC), the terms of reuse of such Open Access shall be governed by a Creative Commons licence – details of these licences and which Creative Commons licence will apply to this Work are set out in our licence referred to above.

Other than as permitted in any relevant BMJ Author's Self Archiving Policies, I confirm this Work has not been accepted for publication elsewhere, is not being considered for publication elsewhere and does not duplicate material already published. I confirm all authors consent to publication of this Work and authorise the granting of this licence.

For peer review only

The Lived Experience Of Participants in an African RandomiseD trial (LEOPARD): Protocol for an in-depth qualitative study within a multi-site randomised controlled trial for HIV-associated cryptococcal meningitis.

David S. Lawrence (david.s.lawrence@lshtm.ac.uk)[1,2†],

Katlego Tsholo (katlegotsholo@yahoo.ie)[2],

Agnes Ssali (agnes.ssali@mrcuganda.org)[3],

Zivai Mupambireyi (zietawana@yahoo.co.uk)[4],

Graeme Hoddinott (graemeh@sun.ac.za)[5],

Deborah Nyirenda (deborahchirwa@gmail.com)[6],

David Meya (david.meya@gmail.com)[7],

Chiratidzo Ndhlovu (mascen@mweb.co.zw)[8],

Thomas S. Harrison (tharriso@sgul.ac.uk)[9],

Joseph N. Jarvis (joseph.jarvis@lshtm.ac.uk)[1,2*] and

Janet Seeley (janet.seeley@lshtm.ac.uk)[1,3*]

[1] Department of Clinical Research, Faculty of Infectious and Tropical Diseases, London School of Hygiene and Tropical Medicine, London, UK

[2] Botswana-Harvard AIDS Institute Partnership, Gaborone, Botswana

[3] MRC/UVRI & LSHTM Uganda Research Unit, Entebbe, Uganda

[4] Centre for Sexual Health and HIV/AIDS Research, Harare, Zimbabwe

[5] Desmond Tutu TB Centre, Department of Paediatrics and Child Health, Faculty of Medicine and Health Sciences, Stellenbosch University, Cape Town, South Africa

[6] Malawi Liverpool Wellcome Trust Clinical Research Programme, Queen Elizabeth Central Hospital, Blantyre, Malawi

[7] Infectious Diseases Institute, Makerere University, Kampala, Uganda

[8] Department of Medicine, University of Zimbabwe College of Health Sciences, Parirenyatwa Hospital, Harare, Zimbabwe

[9] Institute for Infection and Immunity, St George's University of London, and St George's University Hospitals NHS Foundation Trust, London, UK

[†]Corresponding author: david.s.lawrence@lshtm.ac.uk, +267 72464834

[*]Equal contribution

Word count: 3885

For peer review only

ABSTRACT

Introduction: Individuals recruited into clinical trials for life-threatening illnesses are particularly vulnerable. This is especially true in low-income settings. The decision to enrol may be influenced by existing inequalities, poor healthcare infrastructure and fear of death. Where patients are confused or unconscious the responsibility for this decision falls to relatives. This qualitative study is nested in the ongoing AMBIsome Therapy Induction OptimisatioN(AMBITION) trial. AMBITION is recruiting participants from five countries in sub-Saharan Africa and is trialling a novel treatment approach for HIV-associated cryptococcal meningitis, an infection known to affect brain function. We aim to learn from the experiences of participants, relatives and researchers involved in AMBITION.

Methods and Analysis: We will collect data through in-depth interviews with trial participants and the next-of-kin of participants who were confused at enrolment and therefore provided surrogate consent. Data will be collected in Gaborone, Botswana; Kampala, Uganda and Harare, Zimbabwe. Interviews will follow a narrative approach including participatory drawing of participation timelines. This will be supplemented by direct observation of the research process at each of the three recruiting hospitals. Interviews will also take place with researchers from the African and European institutions that form the partnership through which the trial is administered. Interviews will be transcribed verbatim, translated(if necessary), and organised thematically for narrative analysis.

Ethics and Dissemination: This study has been approved by the Human Resource Development Council, Gaborone(HPDME:13/18/1); Makerere School of Health Sciences IRB, Kampala(REF: 2019-061); University of Zimbabwe Joint Research Ethics Committee, Harare(REF: 219/19), and the London School of Hygiene and Tropical Medicine(REF: 17957). Study findings will be shared with research participants from the sites, key stakeholders at each research institution and Ministries of Health to help inform the development and implementation of future trials. The findings of this study will be published in journals and presented at academic meetings.

Trial Registration: Registered at www.clinicaltrials.gov:NCT04296292.

Keywords: HIV; cryptococcal meningitis; clinical trials; informed consent; qualitative research

For peer review only

STRENGTHS AND LIMITATIONS OF THIS STUDY

- There has been no previous qualitative study conducted in a low-income setting which has aimed to explore the experience of individuals who enrol into a clinical trial for the management of a life-threatening illness.
- We plan to collect data from trial participants, their next-of-kin and researchers working on a multi-site clinical trial and by doing this we can elicit a broad range of perspectives and experiences that can inform the improvement of this and similar trials in the future.
- By adopting a multi-site approach, we can compare and contrast experiences across different settings to understand which are shared and which are unique to a particular context.
- The study team are from multiple social and behavioural science disciplines meaning that interpretation of the data will be informed by a range of social theoretical perspectives.
- This study is taking place in a single clinical trial and will collect data from individuals in Botswana, Uganda and Zimbabwe only which means that the results may not be broadly generalisable.

For peer review only - http://bmjopen.bmj.com/site/about/guidelines.xhtml

For peer review only

INTRODUCTION

Since the start of the HIV epidemic our knowledge and understanding of the epidemiology and management of HIV and its numerous complications has exponentially increased. This knowledge has been produced through the conduct of clinical research which would not be possible without the willing consent of participants[1, 2]. Although ART programmes have expanded dramatically and AIDS related deaths have reduced, there were still an estimated 940,000 people who died from AIDS in 2017[3]. In individuals with advanced HIV disease the search for superior treatment options for fatal opportunistic infections continues.

The AMBITION Trial

The AMBIsome Therapy Induction OptimisatioN (AMBITION) trial is a phase-III multi-centred randomised controlled trial recruiting patients with HIV-associated cryptococcal meningitis (CM)[4] (Figure 1). CM is a fungal infection of the brain that occurs most frequently in severely immunocompromised individuals with a CD4 count of less than 100 cells/uL[5]. There are approximately 223,000 incident cases of CM globally, with 73% of these occurring in sub-Saharan Africa. Annual global deaths are estimated at 180,000 and CM is responsible for roughly 15% of all AIDS-related deaths[6]. The nature of the infection means that roughly 40% of patients present with confusion[7] and some with a significantly reduced level of consciousness.

AMBITION is testing a new treatment for CM, a single, high-dose of a less toxic, liposomal form of amphotericin, and is recruiting 850 participants from eight hospitals across five African countries: Botswana, Malawi, South Africa, Uganda and Zimbabwe. The decision making capacity of potential participants is assessed by the clinical team who determine if the individual is able to understand the information around the trial, retain that information, weigh up the information to make a decision and communicate that decision. Patients consent for themselves if deemed to have decision making capacity and if they do not, for example if they are confused or comatose, then a surrogate will do so on their behalf. Participants are followed up daily during their initial inpatient admission (roughly two-weeks) and then fortnightly as an outpatient until they complete the study at ten-weeks. Participants have their medical expenses paid for and receive transport reimbursements to attend outpatient appointments. AMBITION is funded by the European and Developing Countries Clinical Trials Partnership (EDCTP) which brings together researchers from institutions in low-to-middle income countries (LMICs) and Europe.

For peer review only

The AMBITION trial creates a rich environment for an in-depth qualitative study for a number of reasons.

Why participants are motivated to participate in trials: In routine care, mortality with the best standard of care treatment for CM is roughly 50% at 10 weeks[8]. In recent CM trials using the same regimen mortality is roughly 40%[7, 9]. It has been observed that even when using the same drugs as in routine care, trial participants often do better[8]. The reasons for this include having a dedicated research team with more time for patients, better management of drug-induced toxicities and aggressive management of raised intracranial pressure, a common and potentially fatal complication of CM, and, inevitably, some selection of trial participants. Further widening outcomes between routine and trial settings in CM, is the fact that the most effective drugs may be unavailable, or only sporadically available, in routine care. Clinical trials are however designed to answer a research question, the findings of which it is hoped will later be of benefit to a larger population. Some individuals may benefit by participating but it is not designed so that everyone will[10]. Despite this it is not uncommon for research participants to expect a personal therapeutic benefit from the treatment they receive, including in placebo-controlled trials [11, 12]. Other commonly identified motivators are material benefits including free healthcare and transport reimbursements[13-15] but altruism is also a factor[16]. In AMBITION it is fair to expect that all participants will benefit, compared to routine care. What is not understood is how this impacts both patients and researchers when it comes to motivating to enrol in the trial. Their motivation may be rooted in the economic inequality that exists between patient and research institution and which permeate the concept of voluntary participation. Voluntariness is understood as an autonomous choice without material entanglements and the principle of autonomy is often held above others when it comes to consenting for a clinical trial[17]. Research participants who lack agency are therefore subject to 'structural coercion' whereby their social and economic situation drives them into research participation as a means of navigating their illness and because they may not have any other options to get the care they need or desire[18]. This is polarised when the chance of death is high, such as in CM.

Whom to consent when the patient cannot: In the context of life-threatening illness there are questions about when to obtain consent and who to obtain it from. One option is to commence trial procedures and defer consent until the patient is stable, which was acceptable to 70% of parents in a UK-based emergency paediatric study who felt the process was too much to handle in a stressful situation[19]. These findings are consistent with other studies from the UK [20-22]. The Declaration of Helsinki states that it is acceptable to recruit someone without capacity in best interests[23] and it has

For peer review only

been argued that by delaying treatment whilst waiting for consent both risks losing out on the potential health benefit of that specific emergency treatment but also underappreciating the impact of emergency treatment due to systematically delayed initiation[24]. An alternative is therefore to waiver informed consent completely, as was the approach in a post-partum haemorrhage trial in the UK which found that the perceptions of those who gave consent, had a surrogate, or waived consent were not dissimilar [12].

Regarding who provides the consent, it is typical for surrogates to consent on behalf of an unwell patient who is confused or comatose. Within CM studies, roughly 40% of participants are confused and if they regain capacity they re-consent for themselves. Research in HICs has identified that there is generally good concordance between surrogates and patients when it comes to agreeing to consent to both real-life and hypothetical trials but that this is reduced in high-risk trials[25, 26]. In LMICs multiple actors are often involved in the consent process with partners, parents, older family members and community leaders being consulted [11, 27], particularly in the case of severe illness[14]. This extends the process of gaining consent and can delay recruitment and treatment. According to a systematic review of 21 studies in Africa, only 47% of participants undergoing informed consent understood trial procedures such as randomisation and placebo and only 30% were aware they may not experience a therapeutic benefit of participation[28]. Another review found that understanding is significantly diminished amongst those who are critically ill[29]. To date there have been no in-depth qualitative studies in LMICs exploring the process of consent from the perspective of an acutely unwell adult or their consenting next-of-kin.

Participant and Next-of-Kin Experience: We use the broad term of participant experience to encompass the way that an individual navigates through the scheduled events of a clinical trial as detailed in the protocol. Time is a prominent factor throughout this process. An illness occurs at a specific time in someone's life and the entire trial experience is time-bound and shaped by the protocolised schedule of events. A large portion of the ethnographic work exploring participant experience of research in LMICs has elicited data concerning rumours, most commonly blood stealing, which are often dismissed by researchers as expressions of ignorance but are interpreted by social scientists as forms of popular resistance[30-32]. Most ethnographic exploration of rumours has been situated in trials of healthy individuals in trials and less commonly in acute, life-threatening illness. Lumbar puncture, the procedure used to diagnose and treat CM is known to be associated with rumours of causing death[33]. This has not been extensively studied using ethnographic methods but lumbar puncture refusal is common and can be fatal.

For peer review only

In the United States there has been an increasing call to assess clinical trial participant 'patient satisfaction' through the use of surveys or interviews which aim to hear the participant's voice and respond by making local improvement to the trial[34]. In LMICs this approach is less common but the concept of 'good participatory practice' has been developed by the WHO over the years [35] and this involves elements related to the participant experience[36]. No ethnographic work has explored these in the context of acute illness research in sub-Saharan Africa. Research within healthy volunteer studies has found that where poor outcomes such as severe disability or death occur, this has led to the apportioning of blame or the generation of rumours about research studies and institutions[30, 37]. An exploration within AMBITION, where poor outcomes are not uncommon, could provide an opportunity to inform and potentially improve the conduct of this trial and others in the future.

Researcher Experience: Paul Farmer (2002) wrote that 'researcher and subject are living in different worlds'[38] and it is commonly perceived that there is a mismatch between researcher and participant understanding of the research process[30]. Large, randomised controlled trials like AMBITION employ a large number of individuals from different countries[39]. Clinical researchers who interact with individuals and their next-of-kin throughout the trial timeline [15, 27, 40, 41] and a whole range of professionals who are well placed to comment on the research process, regulatory approvals and implementation of a trial and can provide a practical insight and suggestions for improvement [42]. As partners in the research process they can reflect on how clinical trials are conceptualised and designed in addition to the benefits and shortcomings of trans-national partnerships and how we can optimise these relationships for the benefit of participants[43]. International researchers often have a broad range of experience working in clinical trials and can reflect on the evolution of clinical trials over time. As representatives of institutions which are partners (and often the lead) on grant applications, they often help to steer the clinical trial agenda in the region and are well placed to comment on how trials can be improved.

Aim and Objectives

The aim of this study is to explore the experience of participants, their next-of-kin and researchers within the AMBITION trial. By doing this we hope to learn how we can improve the trial experience within AMBITION and future trials for life-threatening illnesses.

Our specific objectives are:

From the perspectives of the participant, next-of-kin and researcher:

For peer review only - http://bmjopen.bmj.com/site/about/guidelines.xhtml

1. To build an understanding of the factors that enhance or diminish a clinical trial experience.

From the perspective of the researcher:

2. To compare the individual researcher's experience of the conceptualisation, development, initiation and implementation of a multi-centred clinical trial in Africa.

METHODS AND ANALYSIS

Study design

An in-depth qualitative study entitled the Lived Experience of Participants in an African RandomiseD trial (LEOPARD). We will adopt a combination of in-depth interviews and direct observations to explore the experience of participants, their next-of-kin and researchers within the AMBITION trial (Figure 2).

Developing of the methodology

The LEOPARD study was conceived by DSL but the methodology was refined with the valuable input of social scientists from each of the six AMBITION trial sites. Each social scientist has a particular interest in clinical trials. Over a series of one-on-one discussions and group calls the LEOPARD study evolved. Having developed a consensus on a methodology it was necessary to determine the feasibility of collecting data from six sites. Recruitment into the LEOPARD study will take place in Gaborone, Harare and Kampala. The reason for limiting data collection to three sites is to enable in-depth data collection and to avoid simply skimming the surface by diluting down data collection across multiple sites. These three sites represent diverse HIV epidemics, healthcare systems and political contexts which can be explored during data analysis.

Conceptual framework

We will use narrative analysis to explore how the concept of time shapes the experience of a life-threatening illness and a clinical trial. Drawing on Nancy Munn's theory of temporalization we will look at how time is experienced by different individuals and how the pressure of a life-threatening illness impacts the perception of time as well as the complex decision to enrol (or not) in a clinical trial. By understanding how time and pressure impact the meaning and understanding of events at a time of crisis, we hope to learn how clinical trials can be better tailored to the needs of individuals with life-threatening illnesses. Narrative analysis is more commonly adopted by studies exploring chronic health conditions but the exploration of time is well suited to narrative analysis and a clinical trial, which has a clearly defined temporal structure, provides a rich setting for storytelling.

For peer review only - http://bmjopen.bmj.com/site/about/guidelines.xhtml

In-depth interviews with AMBITION trial participants

The purpose of the IDIs with AMBITION trial participants is to collect personal accounts of their experience within the trial. Individuals who upon entry into the AMBITION trial were deemed to have decision making capacity (i.e. orientated) and those who were not (i.e. disorientated), and therefore underwent surrogate consent, will be approached. All participants in the LEOPARD study will need to have decision making capacity to contribute to the IDI, meaning that those who lacked decision making capacity at baseline will have clinically improved and regained that capacity. We will aim to recruit a maximum of 20 participants from each of the three sites, 60 in total, with a proposed gender balance of 50-60% male and 40-50% female which is in line with the epidemiology of cryptococcal meningitis at the sites. Consecutively eligible individuals will be approached to participate in two IDIs. One will take place at least six weeks into the ten week AMBITION trial and the other will take place at least four weeks after the trial. The reason for this is to allow reflection on the trial when one is both within and outside of it. Interviews will follow a broad interview schedule and the participant will be invited to draw a timeline of the events before, during and after the trial (Supplementary File 1). If individuals can only contribute to one IDI, for example due to worsening health or unavailability, then the data from the first IDI will be retained and analysed.

In-depth interviews with the next-of-kin of AMBITION trial participants

The purpose of the IDIs with the next-of-kin of AMBITION trial participants is to collect personal accounts from individuals who have cared for and made important decisions about someone with a life-threatening illness. We use the term next-of-kin as a broad umbrella term to include any individual who may be the legal representative, a caregiver, or a surrogate of the participant. This individual will have provided consent for the participant to enrol into the AMBITION trial even if they may not have been the legally defined next-of-kin. We will aim to recruit a maximum of 15 individuals from each site, 45 in total, with no specification for gender. Consecutively eligible individuals will be approached to participate in a single IDI which will take place at least six weeks into the AMBITION trial. At the time of the IDI it will not be necessary for the trial participant to have regained decision-making capacity and these IDIs do not need to be linked to those with participants, although it is anticipated that some will be. Interviews will again follow a broad interview schedule and the participant will be invited to draw a timeline of the events (Supplementary File 2).

In-depth interviews with AMBITION researchers

The purpose of the IDIs with AMBITION researchers is to understand their perspectives on how research is designed and implemented in Africa. Interviews will take place with researchers from each

For peer review only

of the research institutions which form the AMBITION consortium. At African sites where trial participants are being recruited we will approach a range of individuals with different roles including senior and junior researchers, research doctors and nurses, laboratory scientists, pharmacists and study coordinators. In addition, individuals who are based at European institutions will be approached. We will aim for a maximum of 12 individuals from each of the three participating African sites and four from each of the five European sites. The maximum number of researcher interviews will be 56. Individuals will be conveniently sampled and interviewed on a single occasion, following a broad interview schedule (Supplementary File 3).

Direct observations of AMBITION researchers

A period of 12 months will be spend conducting ethnographic fieldwork at the African sites. The objective of this work is to contextualise the data from IDIs within the broader research environment. As the primary focus is on improving the trial for participants, observations will be largely based in the clinical environment, with emphasis placed on observing clinical staff. A total of four researchers from each of the three African sites will be invited to participate in direct observations. It will be made clear that this is not a method designed to appraise an individual, but an opportunity to spend a defined period of time observing events that take place within the research process. Observations will be coupled with brief questions to those in close proximity to the activity under observation.

Principles of recruitment

Eligible individuals will be approached to enrol in the study by a social scientist. In the case of AMBITION trial participants and their next-of-kin, this will be done in the local language by an experienced social scientist at that site. In the case of AMBITION researchers this will be DSL who is part of the AMBITION Trial Management Group in his role as Lead Clinician for the trial. The researcher participant will be assured that they are free to decline participation and are not being interviewed or observed for the purposes of any appraisal or formal evaluation of their role within the team. The purpose of the researcher interviews and observations is to understand the research process and not to criticise individuals. A reflective approach to the research process will be adopted to iteratively refine the data collection methods but also the communication skills of the social scientists.

Eligible individuals will be provided with a Participant Information Sheet and given the opportunity to ask questions. If they agree to participate, they will sign an Informed Consent Form and given the opportunity to withdraw their consent at any time, without giving a reason. Interviews will take place in a mutually acceptable location, be recorded with a digital voice recorder and notes will be taken

For peer review only

during the interview. Observations will not be recorded and field notes will be made after the period of observation has finished.

It is anticipated that this study may identify aspects of the AMBITION trial that need to be improved. In order to ensure this a formal reporting process will be followed. Each of the individual social science research assistants will report back to DSL. Any urgent issues that relate to trial conduct and Good Clinical Practice will be communicated through the use of direct communication and reflective summaries written on the day of data collection. In addition, weekly meetings will take place between the social scientists and DSL to discuss less urgent issues. These findings will be communicated either urgently to the Trial Management Group or at their weekly meetings, whichever is deemed appropriate. Additional advice may be sought from JS who is independent of the AMBITION trial. Following this process the team will determine a course of action which may result, for example, in additional training of trial staff or modification of study procedures. This process is of vital importance to ensure that the findings of this study can improve the conduct of the ongoing AMBITION trial. The confidentiality of the participant will be maintained throughout this process so as not to undermine trust in the study.

Confidentiality

All study documents will be kept on the person of the researcher or in a secure, locked location at all times. All digital documents will be on a password protected, encrypted computer, backed up regularly and only shared with the study team. Names of interviewees will not be used at any stage of the data collection process. Pre-determined identification numbers will be used on data collection forms. Audio recordings will not start until the interviewee has given consent and will not record their name. Pseudonyms will be used throughout. The location of researcher participants will be anonymised because the small number of eligible participants means that stating their location could make it possible to identify them.

Data analysis

Audio recordings will be transcribed verbatim into MS Word, translated into English in a separate second step if necessary, then exported to NVIVO-11 for coding and analysis. The first two IDIs from each group of participants will be analysed and discussed to enable iterative refinement of the data collection approach. Similarly, regular meetings will be used to review data, refine data collection tools and assess for data saturation. We will organise the data thematically and analyse it using narrative analysis at the country level by the social science team at each site. All data from AMBITION

For peer review only - http://bmjopen.bmj.com/site/about/guidelines.xhtml

researchers will be analysed together using thematic analysis which will be performed in six phases: familiarisation with data, initial code generation, searching for themes, reviewing themes, defining and naming themes and presenting final conclusions. These analyses will then be combined in a meta-synthesis of all data, irrespective of location or informant, to identify any areas of disconnect and, by comparing with country specific analyses, to assess generalisability of findings.

Patient and Public Involvement

This protocol has been reviewed by Community Advisory Board members, expert patients and HIV activists from across the African sites. These individuals and groups will continue to be consulted throughout the data collection process and during the dissemination of research findings.

Ethics and Dissemination

This study has been approved by the Human Resource Development Council, Gaborone (HPDME:13/18/1); Makerere School of Health Sciences IRB, Kampala (REF: 2019-061); University of Zimbabwe Joint Research Ethics Committee, Harare (REF: 219/19), and the London School of Hygiene and Tropical Medicine (REF: 17957). Study findings will be shared with research participants from the African and European sites, key stakeholders at each research institution and Ministries of Health to help inform the development and implementation of future trials. The findings of this study will be published in journals and presented at academic meetings.

Authors Contributions: DSL wrote the initial manuscript and trial protocol and is the Chief Investigator for the study. KT, AS and ZM are social scientists based at each of the three African sites. GH is a social scientist in South Africa and contributed to the study design. DN is a social scientist in Malawi and contributed to the study design. DM and CN are the AMBITION and LEOPARD principal investigators at the Kampala and Harare sites respectively. TH and JNJ are co-Chief Investigators of the AMBITION study. JS and JNJ jointly supervise DSL. All authors have been actively involved in the development of this study, the data collection tools and the analysis plan. All authors have reviewed and approved the final manuscript.

Funding statement: This work is funded by the UK National Health Service (NHS) National Institute for Health Research (NIHR), using Official Development Assistant (ODA) funding through a Global Health Professorship to JNJ (Grant RP-2017-08-ST2-012). The views expressed are those of the authors and not necessarily those of the NHS, NIHR, the Department of Health and Social Care, or other funding entities.

For peer review only

Competing Interests: None declared.

Figure 1: AMBITION Study Schema

Figure 2: LEOPARD Study Schema

Supplementary File 1: Participant Interview Schedule

Supplementary File 2: Next-of-kin Interview Schedule

Supplementary File 3: Researcher Interview Schedule

For peer review only

For peer review only - http://bmjopen.bmj.com/site/about/guidelines.xhtml

REFERENCES

1. Broder S. The development of antiretroviral therapy and its impact on the HIV-1/AIDS pandemic. Antiviral research. 2010;85(1):1-18.
2. Epstein S. Democracy, expertise and AIDS treatment activism. In: Kleinman DL, editor. Science, Technology and Democracy. New York, USA: SUNY Press; 2000.
3. UNAIDS. Miles to go - closing gaps, breaking barriers, righting injustices. Global AIDS Update 2018. Geneva: UNAIDS; 2018 13th August 2018.
4. Lawrence DS, Youssouf N, Molloy SF, Alanio A, Alufandika M, Boulware DR, et al. AMBIsome Therapy Induction OptimisatioN (AMBITION): High Dose AmBisome for Cryptococcal Meningitis Induction Therapy in sub-Saharan Africa: Study Protocol for a Phase 3 Randomised Controlled Non-Inferiority Trial. Trials. 2018;19(1):649.
5. Lawrence DS, Youssouf N, Molloy S, Alanio A, Alufandika M, Boulware DR, et al. AMBIsome Therapy Induction OptimisatioN (AMBITION): High Dose Ambisome for Cryptococcal Meningitis Induction Therapy in sub-Saharan Africa: Study Protocol for a Phase 3 Randomised Controlled Non-Inferiority Trial. Trials. 2018;In Press.
6. Rajasingham R, Smith RM, Park BJ, Jarvis JN, Govender NP, Chiller TM, et al. Global burden of disease of HIV-associated cryptococcal meningitis: an updated analysis. The Lancet Infectious diseases. 2017;17(8):873-81.
7. Molloy SF, Kanyama C, Heyderman RS, Loyse A, Kouanfack C, Chanda D, et al. Antifungal Combinations for Treatment of Cryptococcal Meningitis in Africa. The New England journal of medicine. 2018;378(11):1004-17.
8. Tenforde MW, Gertz AM, Lawrence DS, Wills NK, Guthrie BL, Farquhar C, et al. Mortality from HIV-associated meningitis in sub-Saharan Africa: a systematic review and meta-analysis. Journal of the International AIDS Society. 2020;23(1):e25416.
9. Beardsley J, Wolbers M, Kibengo FM, Ggayi AB, Kamali A, Cuc NT, et al. Adjunctive Dexamethasone in HIV-Associated Cryptococcal Meningitis. The New England journal of medicine. 2016;374(6):542-54.
10. Molyneux CS, Peshu N, Marsh K. Understanding of informed consent in a low-income setting: three case studies from the Kenyan Coast. Soc Sci Med. 2004;59(12):2547-59.
11. Leach A, Hilton S, Greenwood BM, Manneh E, Dibba B, Wilkins A, et al. An evaluation of the informed consent procedure used during a trial of a Haemophilus influenzae type B conjugate vaccine undertaken in The Gambia, West Africa. Soc Sci Med. 1999;48(2):139-48.
12. Houghton G, Kingdon C, Dower M, Shakur-Still H, Alfirevic Z. What women think about consent to research at the time of an obstetric emergency: a qualitative study of the views of a cohort of World Maternal Antifibrinolytic Trial participants. BJOG : an international journal of obstetrics and gynaecology. 2018;125(13):1744-53.
13. Corneli AL, McKenna K, Perry B, Ahmed K, Agot K, Malamatsho F, et al. The science of being a study participant: FEM-PrEP participants' explanations for overreporting adherence to the study pills and for the whereabouts of unused pills. Journal of acquired immune deficiency syndromes (1999). 2015;68(5):578-84.
14. Gikonyo C, Bejon P, Marsh V, Molyneux S. Taking social relationships seriously: lessons learned from the informed consent practices of a vaccine trial on the Kenyan Coast. Soc Sci Med. 2008;67(5):708-20.
15. Ssali A, Poland F, Seeley J. Volunteer experiences and perceptions of the informed consent process: Lessons from two HIV clinical trials in Uganda. BMC Med Ethics. 2015;16(1):86.
16. Katz AWK, Mensch BS, Woeber K, Musara P, Etima J, van der Straten A. Understanding women's motivations to participate in MTN-003/VOICE, a phase 2b HIV prevention trial with low adherence. BMC women's health. 2019;19(1):18-.
17. Geissler PW, Kelly A, Imoukhuede B, Pool R. 'He is now like a brother, I can even give him some blood'--relational ethics and material exchanges in a malaria vaccine 'trial community' in The Gambia. Soc Sci Med. 2008;67(5):696-707.

For peer review only

18. Fisher JA. Expanding the frame of "voluntariness" in informed consent: structural coercion and the power of social and economic context. Kennedy Institute of Ethics journal. 2013;23(4):355-79.
19. Gamble C, Nadel S, Snape D, McKay A, Hickey H, Williamson P, et al. What Parents of Children Who Have Received Emergency Care Think about Deferring Consent in Randomised Trials of Emergency Treatments: Postal Survey. PloS one. 2012;7(5):e35982.
20. Kenyon S, Dixon-Woods M, Jackson CJ, Windridge K, Pitchforth E. Participating in a trial in a critical situation: a qualitative study in pregnancy. Quality & safety in health care. 2006;15(2):98-101.
21. Corrigan O. Empty ethics: the problem with informed consent. Sociology of health & illness. 2003;25(7):768-92.
22. Behrendt C, Golz T, Roesler C, Bertz H, Wunsch A. What do our patients understand about their trial participation? Assessing patients' understanding of their informed consent consultation about randomised clinical trials. J Med Ethics. 2011;37(2):74-80.
23. World Medical Association Declaration of Helsinki: ethical principles for medical research involving human subjects. Jama. 2013;310(20):2191-4.
24. Roberts I, Prieto-Merino D, Shakur H, Chalmers I, Nicholl J. Effect of consent rituals on mortality in emergency care research. Lancet (London, England). 2011;377(9771):1071-2.
25. Newman JT, Smart A, Reese TR, Williams A, Moss M. Surrogate and patient discrepancy regarding consent for critical care research. Critical care medicine. 2012;40(9):2590-4.
26. Coppolino M, Ackerson L. Do surrogate decision makers provide accurate consent for intensive care research? Chest. 2001;119(2):603-12.
27. Kingori P. Experiencing everyday ethics in context: frontline data collectors perspectives and practices of bioethics. Soc Sci Med. 2013;98:361-70.
28. Afolabi MO, Okebe JU, McGrath N, Larson HJ, Bojang K, Chandramohan D. Informed consent comprehension in African research settings. Tropical Medicine & International Health. 2014;19(6):625-42.
29. Tam NT, Huy NT, Thoa le TB, Long NP, Trang NT, Hirayama K, et al. Participants' understanding of informed consent in clinical trials over three decades: systematic review and meta-analysis. Bulletin of the World Health Organization. 2015;93(3):186-98h.
30. Fairhead J, Leach M, Small M. Where techno-science meets poverty: medical research and the economy of blood in The Gambia, West Africa. Soc Sci Med. 2006;63(4):1109-20.
31. Geissler PW. 'Kachinja Are Coming!': Encounters around Medical Research Work in a Kenyan Village. Africa: Journal of the International African Institute. 2005;75(2):173-202.
32. Geissler PW, Pool R. Editorial: Popular concerns about medical research projects in sub-Saharan Africa--a critical voice in debates about medical research ethics. Tropical medicine & international health : TM & IH. 2006;11(7):975-82.
33. Thakur KT, Mateyo K, Hachaambwa L, Kayamba V, Mallewa M, Mallewa J, et al. Lumbar puncture refusal in sub-Saharan Africa: A call for further understanding and intervention. Neurology. 2015;84(19):1988-90.
34. Pflugeisen BM, Rebar S, Reedy A, Pierce R, Amoroso PJ. Assessment of clinical trial participant patient satisfaction: a call to action. Trials. 2016;17(1):483.
35. McGrory E, Irvin A, Heise L. Research Rashomon: Lessons from the Cameroon Pre-exposure Prophylaxis Trial Site. Washington DC: Global Campaign for Microbicides at PATH; 2009.
36. Mack N, Kirkendale S, Omullo P, Odhiambo J, Ratlhagana M, Masaki M, et al. Implementing good participatory practice guidelines in the FEM-PrEP Preexposure Prophylaxis Trial for HIV Prevention among African Women: a focus on local stakeholder involvement. Open Access Journal of Clinical Trials. 2013;5:127-35.
37. Geissler PW. 'Transport to Where?': Reflections on the problem of value and time a propos an awkward practice in medical research. J Cult Econ. 2011;4(1):45-64.
38. Farmer P. Can transnational research be ethical in the developing world? Lancet (London, England). 2002;360(9342):1266.

For peer review only - http://bmjopen.bmj.com/site/about/guidelines.xhtml

39. Molyneux S, Geissler PW. Ethics and the ethnography of medical research in Africa. Soc Sci Med. 2008;67(5):685-95.
40. Kingori P, Gerrets R. Morals, morale and motivations in data fabrication: Medical research fieldworkers views and practices in two Sub-Saharan African contexts. Soc Sci Med. 2016;166:150-9.
41. Monroe A, Nakigozi G, Ddaaki W, Bazaale JM, Gray RH, Wawer MJ, et al. Qualitative insights into implementation, processes, and outcomes of a randomized trial on peer support and HIV care engagement in Rakai, Uganda. BMC infectious diseases. 2017;17(1):54.
42. Vischer N, Pfeiffer C, Limacher M, Burri C. "You can save time if..."-A qualitative study on internal factors slowing down clinical trials in Sub-Saharan Africa. PloS one. 2017;12(3):e0173796.
43. Franzen SRP, Chandler C, Enquselassie F, Siribaddana S, Atashili J, Angus B, et al. Understanding the investigators: a qualitative study investigating the barriers and enablers to the implementation of local investigator-initiated clinical trials in Ethiopia. BMJ open. 2013;3(11):e003616.

For peer review only

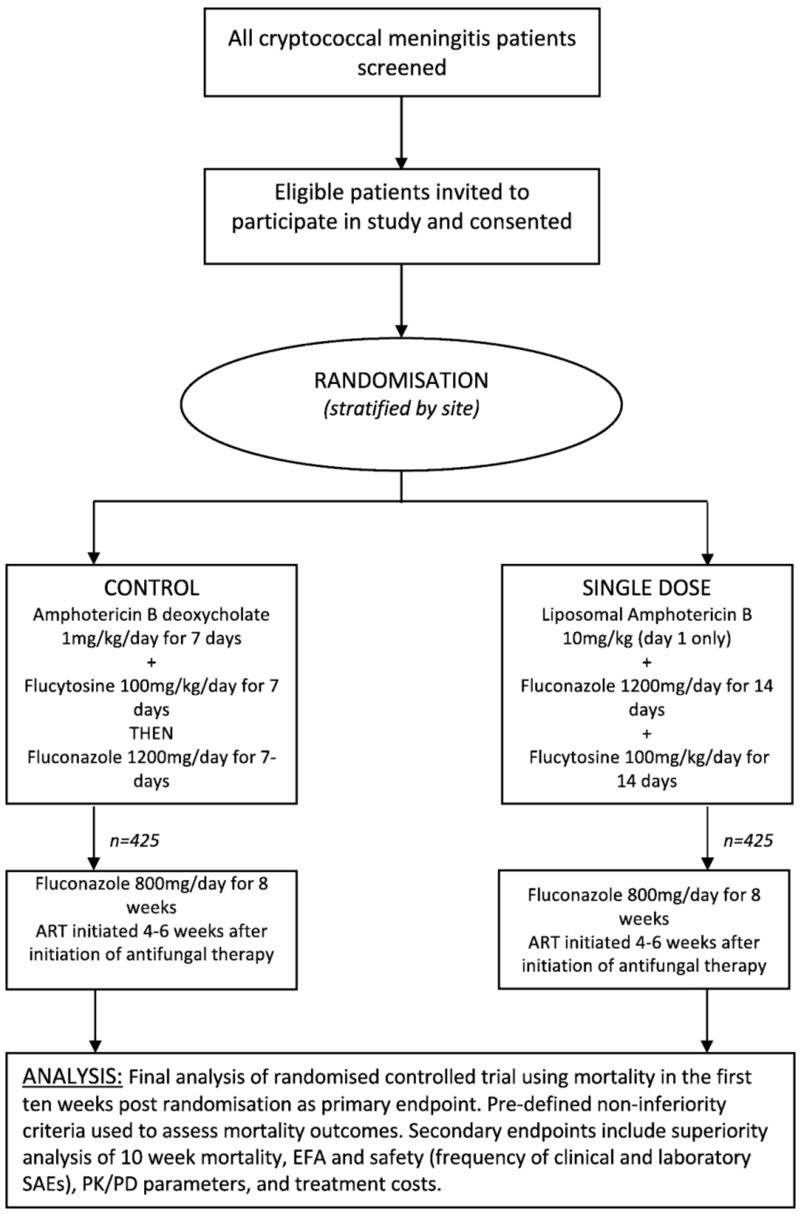

AMBITION Study Schema

417x633mm (72 x 72 DPI)

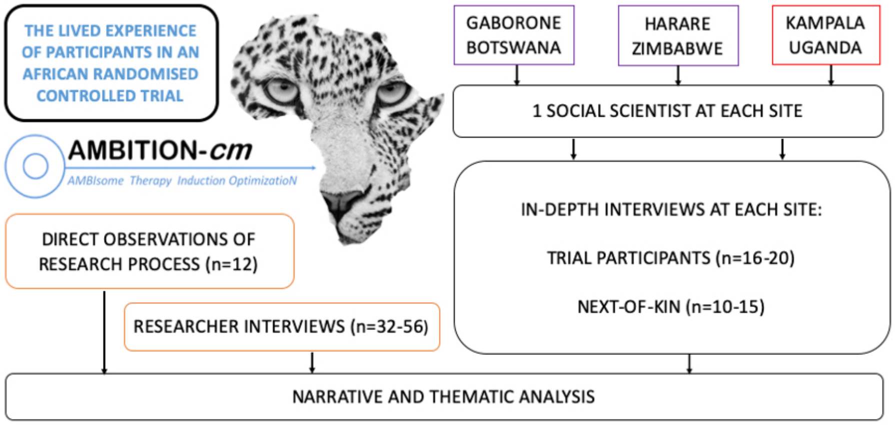

LEOPARD Study Schema

348x179mm (70 x 70 DPI)

The Lived Experience Of Participants in an African RandomiseD controlled trial (LEOPARD)

Participant In-depth Interview Schedule

Note: This is purely a guide for a semi-structured interview and is not a rigid script. The interview should attempt to cover the key themes of enquiry outlined below but the participant should be able to steer the conversation and deviate from these themes if desired.

Introduction:

- General purpose and overview of the study
- Aims of interview
- Why the participant's cooperation is important
- Assurance of confidentiality
- What will happen with the collected information
- Any questions?
- Consent

'The aim of this exercise and series of questions is to understand a little about you and to hear your experience of the trial process from before you were recruited, the consent process, and throughout the trial itself'

Demographics and Background

- Age
- Gender
- Occupation

'At this point I would like you to consider drawing your experience in the trial as a timeline onto this piece of paper. I would like to know how you experienced each of the parts of the trial, one after the other, from just before you joined the trial until today.'

The participant can decline this approach if they wish. If they do want to draw a timeline let them direct the conversation and try to understand their recollection of events. Use prompts to ask follow-up questions as suggested below.

Before the study

- Previous experience with clinical trials, if any
- Previously held perceptions of clinical trials
- General health
- Circumstances in which participant became unwell
- How dis/orientated they were, their recollection of events
- The admission to the hospital, including experience of diagnostic lumbar puncture
- Diagnosis of cryptococcal meningitis and any other illness

Recruitment

- Experience of being approached by the team

For peer review only - http://bmjopen.bmj.com/site/about/guidelines.xhtml

- First impressions of the clinical trial
- Thoughts on the participant information sheet

Consent

- How did they decide
- What was their motivation and what were their main concerns
- How long did it take to decide
- Did they feel under pressure to consent and if so, by whom
- With whom did they decide
- Did someone decide on their behalf and if so, what is their recollection of that and how did they feel both around that time and now
- When completing the form did they feel that they knew what they were signing up for
- Is there any way this process could have been improved

Within the trial (inpatient)

- Was anything different after they entered the trial compared to before
- What did they think about the nature, number and frequency of the procedures they had e.g. blood tests and lumbar punctures
- What did they think about the drugs they were receiving particularly the night time doses
- Are there any specific experiences whilst in hospital they would like to discuss
- Were they confused by what was going on at any point
- How was the communication and care from the trial team

Within the trial (outpatient)

- How was the outpatient clinic and did you have any concerns (such as confidentiality, security, cleanliness)
- How was your experience of those outpatient visits
- Did they miss any appointments during the trial and if so, why
- At any time did they consider leaving the trial and if so, why
- What did they think about the transport reimbursement, was it enough, did it play a role in encouraging them to attend outpatient visits
- How did they feel being asked the health economics questions
- Can you summarise the AMBITION trial

For participants that were confused, ask these questions at appropriate moments

- Did they understand what was happening
- If not, when did they begin to understand what was happening
- Did their confusion resolve all at once or did it come and go
- When they were informed they were in a clinical trial, what were their thoughts
- Who provided consent for them when they were confused, have they discussed this with the person/people and how do they feel about this now

For participants that have completed the study, ask these questions at appropriate moments

- How do they feel now they have left the trial
- Have they been back to their usual care provider and if so, how was that experience

For peer review only - http://bmjopen.bmj.com/site/about/guidelines.xhtml
For peer review only

- What would they like to have seen done differently within the course of the trial
- If they were approached to take part in a clinical trial in the future what would they do and why

Closing:

Is there anything else you think is important that we have not talked about?

- Summarise
- Thank participant
- Provide contacts to participant

Second interview

A second interview will take place after the participant has exited the AMBITION study. During the second interview, spend time reviewing the information that was captured in the first and asking the participant if they have changed how they feel since exiting the study.

Any aspects of this interview schedule that were not captured in the first interview can be addressed in the second.

For peer review only - http://bmjopen.bmj.com/site/about/guidelines.xhtml

AMBITION-cm
AMBIsome Therapy Induction OptimizatioN

LEOPARD

The Lived Experience Of Participants in an African RandomiseD controlled trial (LEOPARD)

Next-of-kin In-depth Interview Schedule

Note: This is purely a guide for a semi-structured interview and is not a rigid script. The interview should attempt to cover the key themes of enquiry outlined below but the participant should be able to steer the conversation and deviate from these themes if desired.

Introduction:

- General purpose and overview of the study
- Aims of interview
- Why the participant's cooperation is important
- Assurance of confidentiality
- What will happen with the collected information
- Any questions?
- Consent

'The aim of this exercise and series of questions is to understand a little about you and to hear your experience as the next-of-kin of someone who was recruited into the AMBITION trial. We are interested to hear your experience of the trial process for your loved one from before they were recruited, the consent process, and throughout the trial itself'

Demographics and Background

- Age
- Gender
- Occupation

'At this point I would like you to consider drawing your experience with the trial as a timeline onto this piece of paper. I would like to know how you experienced each of the parts of the trial, one after the other, from just before you were aware of the trial until today.'

The next-of-kin participant can decline this approach if they wish. If they do want to draw a timeline let them direct the conversation and try to understand their recollection of events. Use prompts to ask follow-up questions as suggested below.

Before the study

- Previous experience with clinical trials, if any
- Previously held perceptions of clinical trials
- Circumstances in which the trial participant became unwell
- How dis/orientated they felt to be at the time
- The admission to the hospital, including experience of diagnostic lumbar puncture
- How and if they were informed of the diagnosis of cryptococcal meningitis and any other illness

Recruitment

For peer review only - http://bmjopen.bmj.com/site/about/guidelines.xhtml
For peer review only

- Experience of being approached by the team
- First impressions of the clinical trial
- Thoughts on the participant information sheet

Consent (Next-of-kin perspective)

- How did they decide
- What was their motivation and what were their main concerns
- How long did it take to decide
- Did they feel under pressure to consent and if so, by whom
- With whom did they decide
- When completing the form did they feel that they knew what they were signing their loved one up for
- Is there any way this process could have been improved

Consent (Participant perspective)

- Did they think their loved one understood what was happening
- If not, when did they begin to understand what was happening
- Did their confusion resolve all at once or did it come and go
- When they were informed they were in a clinical trial, were they part of the re-consent process
- Have they ever discussed this issue of consent with their loved one and if so would they be willing to share this discussion with the researcher

Within the trial (inpatient)

- Was anything different after the participant entered the trial compared to before
- What did they think about the nature, number and frequency of the procedures their loved one had e.g. blood tests and lumbar punctures
- What did they think about the drugs they were receiving particularly the night time doses
- Are there any specific experiences whilst in hospital they would like to discuss
- Were they confused by what was going on at any point
- How was the communication and care from the trial team
- Did they feel that they were involved in the trial process

For the next-of-kin that accompanied the participant to outpatient appointments, ask these questions at appropriate moments

- How was the outpatient clinic and did you have any concerns (such as confidentiality, security, cleanliness)
- How was your experience of those outpatient visits
- Did your loved one miss any appointments during the trial and if so, why
- At any time did they consider removing their loved one from the trial and if so, why
- At any time did their loved one consider removing themselves from the trial and if so, why
- What did they think about the transport reimbursement, was it enough, did it play a role in encouraging their loved one to attend outpatient visits and for them to accompany them
- Can they summarise the AMBITION trial

For peer review only - http://bmjopen.bmj.com/site/about/guidelines.xhtml

Reflections on the trial

- How do they feel about the trial in general
- What would they like to have seen done differently within the course of the trial
- If they were approached to take part in a clinical trial in the future what would they do and why

Closing:

Is there anything else you think is important that we have not talked about?

- Summarise
- Thank participant
- Provide contacts to participant

For peer review only

For peer review only - http://bmjopen.bmj.com/site/about/guidelines.xhtml

AMBITION-cm
AMBIsome Therapy Induction OptimizatioN

LEOPARD

The Lived Experience Of Participants in an African RandomiseD controlled trial (LEOPARD)

Researcher In-depth Interview Schedule

Note: This is purely a guide for a semi-structured interview and is not a rigid script. The interview should attempt to cover the key themes of enquiry outlined below but the participant should be able to steer the conversation and deviate from these themes if desired.

Introduction:

- General purpose and overview of the study
- Aims of interview
- Why the participant's cooperation is important
- Assurance of confidentiality
- What will happen with the collected information
- Any questions?
- Consent

'The aim of the first series of questions is to contextualise you within the Ambition study and the clinical research community'

Demographics and Background:

- Job title and role
- Institution and number of years there
- Training / qualifications and their locations
- Research posts previously held and their locations

The Ambition study:

- How became involved
- Current role and responsibilities
- Level of engagement with trial participants

Previous research experience:

- Background of working with participants of other trials
- With individuals within Ambition and/or not affiliated
- In other institutions
- In transnational research partnerships

'Drawing predominantly on your current experience within the Ambition study but also from your previous work (if any) please can you share your thoughts on the following:'

Trial participant experience:

- General impressions of how participants experience a trial
- What you and fellow researchers are good at
- What you are not so good at
- Experience of evaluating trial participant experience

For peer review only - http://bmjopen.bmj.com/site/about/guidelines.xhtml

- Suggestions for improvements

Specifically aim to focus on: the consent process, recruiting participants with impaired consciousness and the death of participants, prompting if required. Probing to draw on specific examples to elicit narratives.
'Do you think that the issues you have brought up are specific to your hospital / institution / city / country. Where else can you see they do / may occur?'

Transnational research partnerships:

- Understanding of the EDCTP and how it works
- Perceived benefits of such an approach
- Any shortcomings
- Capacity building
- Ownership
- Impact on the global research agenda
- Any suggestions for improvement

'Do you think that the issues you have brought up are specific to your hospital / institution / city / country. Where else can you see they do / may occur?'

Closing:

Is there anything else you think is important that we have not talked about?

- Summarise
- Thank participant
- Provide contacts to participant

For peer review only - http://bmjopen.bmj.com/site/about/guidelines.xhtml
